# Culture-free perceptual invariant for trustworthiness

**DOI:** 10.1371/journal.pone.0263348

**Published:** 2022-02-10

**Authors:** Ce Mo, Irene Cristofori, Guillaume Lio, Alice Gomez, Jean-René Duhamel, Chen Qu, Angela Sirigu

**Affiliations:** 1 Department of Psychology, South China Normal University, Guangzhou, China; 2 Institute of Cognitive Sciences Marc Jeannerod CNRS, UMR 5229, Bron, France; 3 University of Lyon, Villeurbanne, France; 4 iMIND Center of Execellence for Autism, Le Vinatier Hospital, Bron, France; University Hospitals Tubingen: Universitatsklinikum Tubingen, GERMANY

## Abstract

Humans beings decide to trust others selectively, often based on the appearance of a face. But how do observers deal with the wide variety of facial morphologies and, in particular, those outside their own familiar cultural group? Using reverse correlation, a data-driven approach to explore how individuals create internal representations without external biases, we studied the generation of trustworthy faces by French and Chinese participants (N = 160) within and outside their own cultural group. Participants selected the most trustworthy or attractive (control condition) face from two identical European or Asian descent faces that had been modified by different noise masks. A conjunction analysis to reveal facial features common to both cultures showed that Chinese and French participants unconsciously increased the contrast of the "pupil-iris area" to make the face appear more trustworthy. No significant effects common to both groups were found for the attraction condition suggesting that attraction judgements are dependent on cultural processes. These results suggest the presence of universal cross-cultural mechanisms for the construction of implicit first impressions of trust, and highlight the importance of the eyes area in this process.

## 1. Introduction

Trusting others is a pervasive need in humans, as it is essential to an individual’s survival [1]. In our modern societies, trust has become even more important than in the past, as many of the decisions we make are based on trusting strangers (e.g., trusting the representative acting on one’s behalf in government, in science, in education, etc.). So, in many real-life contexts, we trust others without the benefit of our past experiences.

A large body of literature has shown that under uncertainty, people generate feelings of trust based on the appearance of a face [2–5]. This is called the first impression and it corresponds to a kind of subliminal representation of trust associated with the processing of specific facial cues, generated quickly and outside any conscious decision [6–8]. The facial cues that contribute to trust first impression include a smooth, small face, a smiling mouth, opened eyes [9] and a low face width to height ratio [10].

The use of facial morphology to determine trustworthiness raises the question of how observers deal with the wide variety of facial features and especially those that are not part of the cultural group they belong and they are used to. Indeed, it is still unclear whether attributes related to trustworthiness based on facial evaluation are culture-specific or not. In addition, there is no consensus on whether these intercultural facial attributes are specific to evaluations of trust or whether evaluations of other traits such as attractiveness, competence or dominance may involve similar processes.

Studies supporting the socio-cultural dependency hypothesis have shown that trust judgments are influenced by typicality. Typical faces are retrieved from previously seen faces, suggesting that they are perceived as more trustworthy because they recall past experiences [11–13].

Cultural artifacts such as television, books, and visual media expose individuals to specific representations of what looks like a trustworthy and untrustworthy face. Trustworthiness judgements inferred from facial features could therefore be culturally biased or constructed through statistical learning [14]. A study by Todorov’s group showed that when participants make explicit trust judgments, they tend to prefer faces typical of their own culture [14]. However, this study did not specifically address the existence of culture-free facial cues involved in trust first impression. Given the central role of trust in social exchanges and its implications for individuals well-being, one would expect to find mechanisms for assessing trust based on facial features that are not culturally related, which would be of great relevance for many situations that people encounter in our modern society (tourists asking for help when traveling abroad or newly emigrated populations whose adaptation requires the rapid formation of trust bonds).

Surprisingly, there is little direct evidence to support the culture-free first impression hypothesis, although some studies point in this direction. For instance, infants have been shown to prefer trustworthy faces after only a few months of life [15]. Similarly, macaque monkeys gaze significantly longer at trustworthy human faces compared to the untrustworthy ones, as do humans [16].

Here, we examined the following questions. (1) Is the first impression of trustworthiness a culturally independent representation? Are there universal facial features that play a key role in processing trust for in-group and out-group individuals?

In this study we investigated how French (of European origin) and Chinese participants judged trustworthiness of European (in-group and out-group) and Asian (out-group and in-group respectively) faces. During the task, participants saw two pictures of two identical European or Asian faces that had been modified by different noise masks, and had to decide which was more trustworthy. To assess the specificity of facial processing mechanisms linked to the perception of trustworthiness we also tested attractiveness judgements as a control condition.

To model trust during face evaluation, we used a reverse correlation paradigm, a unique data-driven approach allowing the exploration of how individuals build internal visual representations (in this study, trustworthiness/attractiveness). Oosterhof and Todorov [17] demonstrated that by using reverse correlation methods in social evaluation protocols, subjects spontaneously form internal social representations without relying on researchers’ a priori assumptions about those representations (i.e., unbiased stimuli).

This method involves generating two pictures from an original face stimulus through the addition and subtraction of a noise mask. Participants are asked to make a first impression judgement by selecting the item that appears to be most representative of the relevant dimension (in this case, trustworthiness or attractiveness). Thus, both images contain the same face, but the added noise mask alter the appearance in a way that could enhance or reduce the perception of the relevant dimension in relation to the individual internal state. Participants’ decisions are thus determined solely by the variation in noise present in the stimulus [18, 19]. (see **S1 Fig in S1 File** and Methods for details about stimulus generation and testing procedure). Using participants’ choices and the corresponding noise masks, we can then compute a model, the so-called classification images (CIs), reflecting the mental representation of interest. To show cross-cultural processing of facial cues important for trust evaluation, we performed a conjunction analysis to reveal the strategic choices of face features for trust and attractiveness domains common to both European and Asian participants.

## 2. Method

### 2.1 Participants

Eighty Chinese students of Asian origin (40 F) recruited from the South China Normal University, (mean age = 20.71, SD = 1.71) and eighty French students of European origin (40 F) from the University of Lyon 1 Claude Bernard (Mean age = 22.72, SD = 4.22) completed the study. Participants received monetary compensation for their participation (25 CNY, 5 Euros).

**Fig 1:**
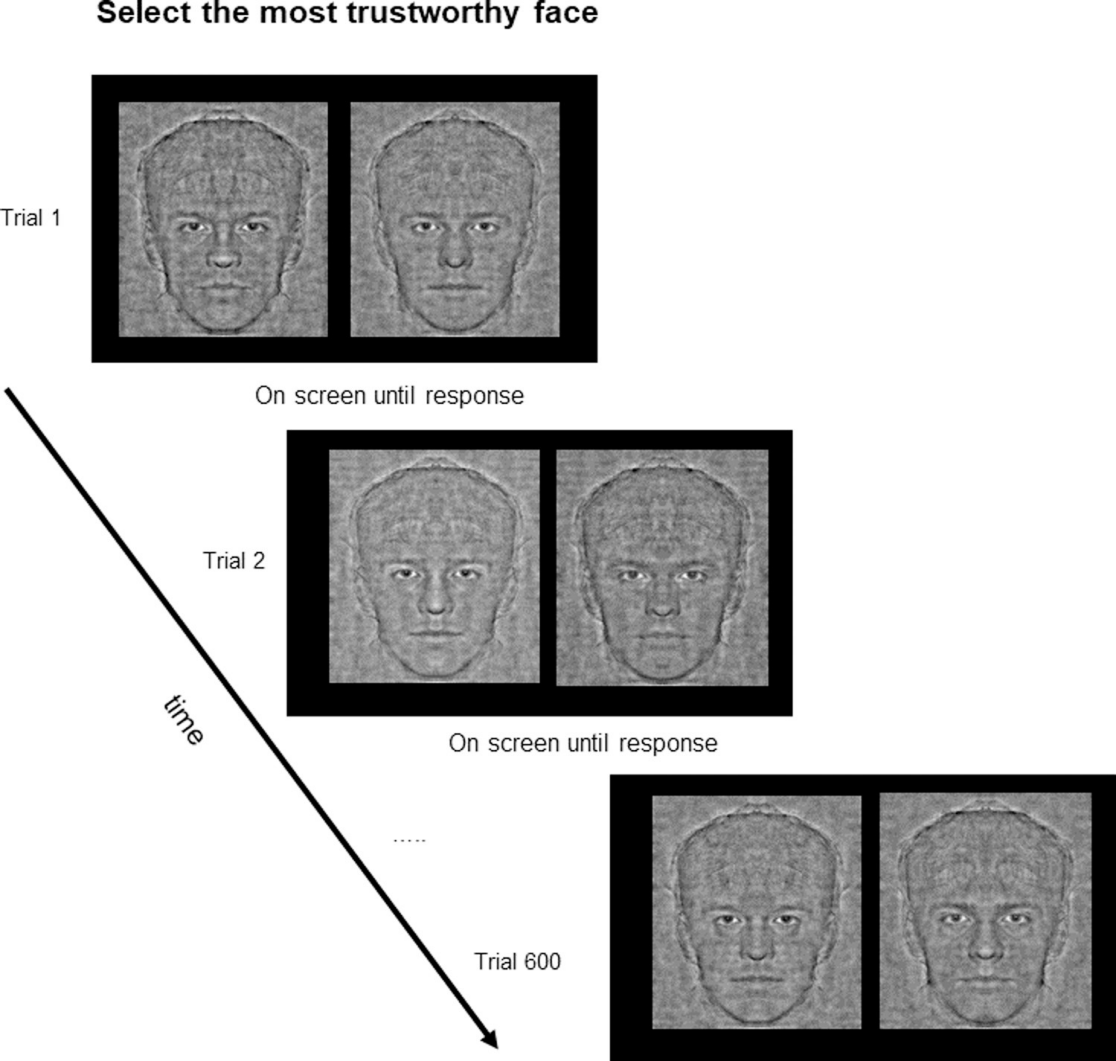
Experiment design. Each participant performed trials from both an in-group and an out-group condition (600 trials). In other words, French [Chinese] participants performed the task evaluating European descent [Asian] and Asian [European descent] faces, respectively in-group and out-group conditions. A control experiment was used to evaluate in-group and out-group attractiveness.

Each participant completed two Reverse Correlation tasks (one with a prototypical Asian face and one with a prototypical European descent face) and was randomly assigned to the trust or attractiveness judgment condition. In essence, each participant made choices of trust on Asian and European descent faces or attractiveness on Asian and European faces. The order of presentation of Asian and European descent faces was counterbalanced. See **Fig 1** for the experimental design. The study was approved by ethical committees in France (CPP Lyon Sud Est) and in China (South China Normal University, Guangzhou). Participants signed written informed consent before study participation.

### 2.2 Reverse correlation task

Trustworthiness and attractiveness judgments were investigated using the forced choice variant of two-image in Mangini and Biederman’s reverse correlation (RC) task [20]. In the RC task, participants were asked to classify replicas of the same face overlaid with different noises. Participants were unaware that the underlying face was identical throughout the task. At the end of the experiment, a model of the participant’s internal representation called the *classification image* (CI) was obtained by averaging all selected noise patterns and adding the result to the original prototypical face image. Modifications were applied to the original task regarding (1) the construction of the neutral basal faces and (2) the generation of the noise masks.

### 2.3 Face stimuli

To identify facial cues that were consistently modulated across the two populations tested, we computed two prototypical neutral faces, one for each ethnic group, using the following procedure. First, we selected six male neutral faces (three European and three Asian descent) from two databases that were controlled for orientation, position and light exposure [Nimstim database [21] for the European descent faces, and an in-lab built database for Asian faces]. We used only male faces to eliminate variability in judgment due to interaction with gender (*47*). Second, each image was digitally edited with the GIMP software (http://gimp.org) according to the following specifications: image size (600x737 pixels), inter-ocular distance (176 pixels), left eye location (x = 212, y = 348 from the lower left corner), eyes-nose distance (100 pixels), eyes-mouth distance (193 pixels), eyes-chin distance (332 pixels), perfect vertical symmetry by averaging the original picture with their flipped left-right counterpart. Third, edited images of the same ethnic group were averaged to obtain a prototypical neutral Asian or European descent face. Fourth, the two prototypical faces were subjected to high-pass filtering by removing their low frequency component calculated with a Gaussian smoothing filter (standard-deviation σ = 10 pixels–filter size = 6.σ). Finally, the luminance/contrast between the two final images were matched using a histogram-equalization procedure (see **S1 Fig in S1 File**). Stimuli constructed in this manner require fewer trials to obtain reliable results compared to the traditional reverse correlation procedure according to our preliminary results. By controlling the proportions and symmetry of the faces, the three basic images were sufficient to create a prototypical identity while avoiding losing important details in the structure of the face (jawbone, nose shape, mouth shape and eye shape).

### 2.4 Generation of the noise masks

Most reverse-correlation experiments use a noise generation procedure that involves superimposing truncated sinusoid patches with different phases, orientations and scales. This procedure generates noise with a frequency content similar to that of the base image making it easier for relevant face modulations to appear during the experiment [9]. Here, we used a different procedure but with the same objectives. First, we transposed the base image into a frequency space using the 2D Fast Fourier Transform. Second, we randomized the phase information and generated the noise mask using inverse Fast Fourier Transform. Using this protocol, the 2D power spectral densities of the noise masks were constant and matched with the 2D power spectral density of the base face image. Finally, as with the prototypical faces, the noise mask was generated by adding vertical symmetry as an additional constraint. This last point was included for several reasons: (1) to preserve the statistical power of the analysis by avoiding testing an unnecessary large number of pixels [22], (2) to reduce trivial modulations of the attractiveness factor caused by asymmetric modulations of the faces [for a review see [23]] and (3) to reduce trivial modulations of the trust and attractiveness factors caused by strong modulations of faces typicality.

### 2.5 Design and procedure

In each trial, two stimuli were presented side by side. One stimulus was a prototypical face with a randomly generated noise mask superimposed on it (signal/noise ratio = 1) and the other was the same prototypical face but with the negative version of the same generated noise mask. Each participant completed two blocks of the RC task each consisting of 600 trials per condition (Asian and European descent faces), i.e., each participant completed 1200 trials in total. For both blocks, participants were asked to select on each trial, the most trustworthy or the most attractive face. Participants were asked to provide only one judgment (i.e., trustworthiness or attractiveness) to avoid psychological bias from one condition to the another. In addition, to avoid response bias inherent with negative social judgments [24] and encourage quick responses based on first impressions, only judgments with a positive valence were requested (**Fig 1**).

### 2.6 Classification images

**Fig 2:**
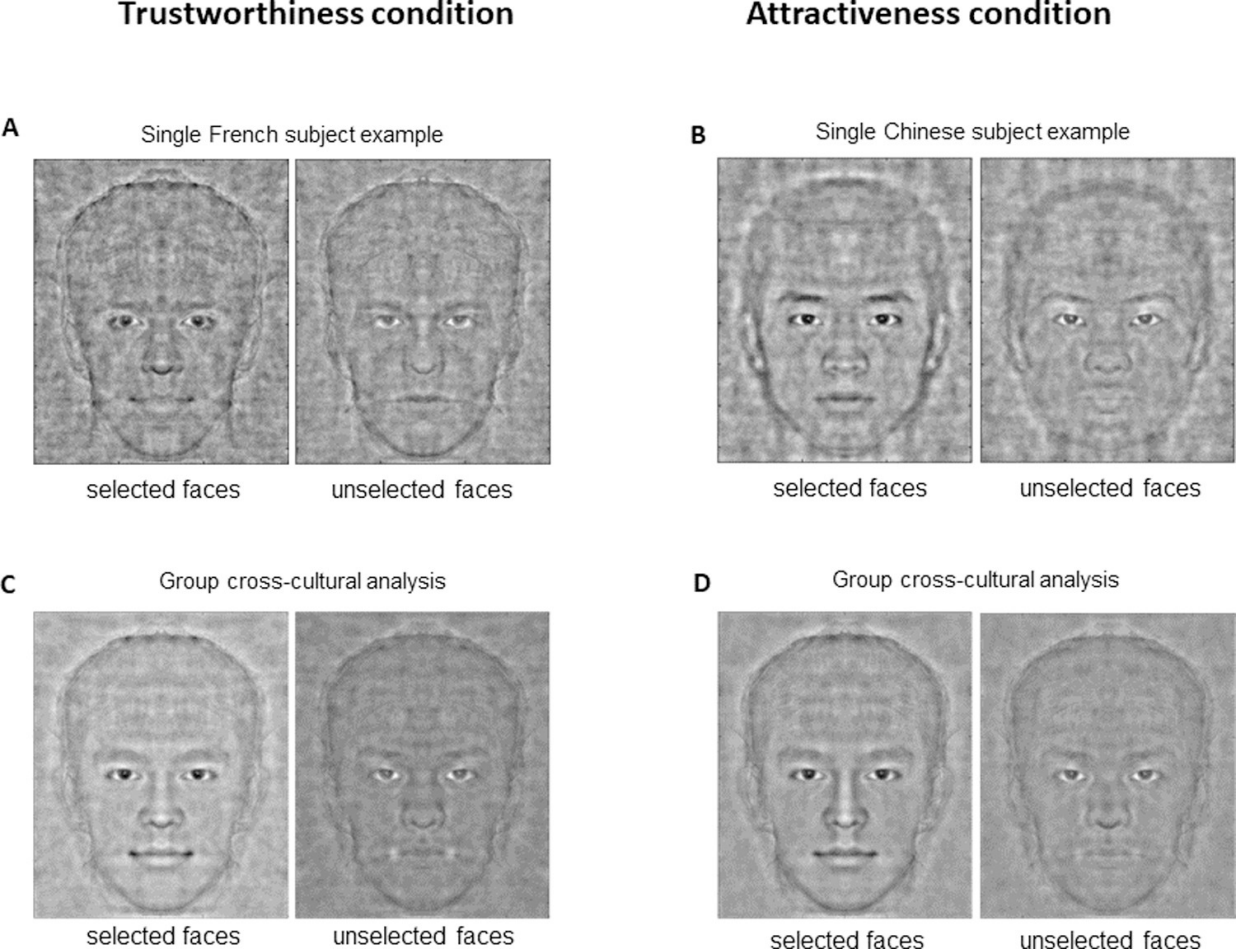
Classification images. Classification images were obtained by averaging the noise patterns selected by the subject and by adding or removing the obtained averaged noise from the original face stimulus. By doing so, the consistently modulated face parts were highlighted. **A and B–Single subject classification images.** (**A**) Example of a single subject classification image (signal/noise ratio = 1) of one French participant for a judgment of trust on the European descent stimulus. (**B**) Example of a single subject classification image (signal/noise ratio = 1) of one Chinese participant for a judgment of attractiveness on Asian faces. For each participant, the reverse correlation task reveals a picture with important modulations of the facial cues and the face structures of the baseline stimulus, revealing an image of each subject’s internal representation linked to their judgment. **C and D–Mean classification images.** (**C**) Grand average of all 160 classification images (European and Asian descent faces) obtained from the all 80 participants (French and Chinese) who made trustworthiness judgments (signal/noise ratio = 1/3). (**D**) Grand average of all 160 classification images (European and Asian descent faces) obtained from all 80 participants (French and Chinese) who made an attractiveness judgment (signal/noise ratio = 1/3). By doing so, pixels that are consistently modulated at the population level are highlighted while subject-specific and culture-specific modulations are averaged out.

At the single-subject level, we computed classification images (CIs) by averaging all noise patterns that a participant had selected as the most trustworthy (or attractive). For visualization purposes, the averaged noise pattern was overlaid on to the appropriate prototypical face image with a signal-to-noise ratio of 1. At the group level, we Z-transformed all single-subject CIs and averaged them to produce a group CI. For visualization, we overlaid the group CI with a signal-to-noise ratio of 1/3. We reduced the signal-to-noise ratio because, at the group level, the variance of the group CI was greatly reduced since only significantly modulated areas differed from 0. An example of single subject CI is shown in **Fig 2A** and **2B** for the trust and attractiveness condition.

### 2.7 Statistical analyses

#### 2.7.1 Group-level analysis

For each task, we identified significantly modulated facial regions using a non-parametric cluster-based permutation test (p<0.05, Family Wise Error Rate corrected for multiple comparisons) comprising 1000 replications of the task with random choices. Given the symmetry of the generated noise, only pixels on one side of the face (including hair) were tested. We produced four group-level mean CI images for trustworthiness and for attractiveness (mean CI 1: from the 40 French subjects’ CI of the European descent stimuli; mean CI 2: from the 40 French subjects’ CI of the Asian stimuli; mean CI 3: from the 40 Chinese subjects of the European descent stimuli; mean CI 4: from the 40 Chinese subjects of the Asian stimuli). Finally, for illustrative purposes (see **Fig 2C** and **2D**), we also computed a group-level mean CI of 160 images of the two stimuli types (Asian and European descent) of the CI of each of the 80 subjects’ (40 French, 40 Chinese) for each judgment (trust and attractiveness).

#### 2.7.2 Conjunction analysis

To identify pixels that were consistently modulated across different populations and stimuli, we applied three conjunction analyses per condition (trustworthiness or attractiveness): (1) within-group analyses (conjunction of experiments where participants of an ethnic group rate faces from the same ethnic group, mean CI 1 and 3); (2) between-group analysis (conjunction of the experiments where participants evaluate faces from another ethnic group, mean CI 2 and 4); (3) Analysis of all subjects (conjunction of all experiments with the same face evaluation, mean CI 1, 2, 3 and 4 –trust or attractiveness). Only significant pixels modulated consistently across all examined experiments were considered significant. In a conjunction analysis between different experimental conditions, a pixel is considered significant if it is significant for each of the different conditions. Here, a pixel is significant if it is either significantly darkened under the different conditions, or if it was significantly lightened under the different conditions. For visualization, dark pixels that were significantly more selected than light pixels are represented with a blue color scale and light pixels that were significantly more selected than dark pixels are represented by a red color scale. The value represented in the color scale corresponds, for each pixel, to the minimal absolute value of the significant t-statistics.

Statistical analyses were carried out using Matlab r2018a (MathWorks; Natick, MA). Data were tested for Gaussian distribution (Lilliefors test) and variance homogeneity (Fisher tests). Unless otherwise specified, data were normally distributed, and assumptions for analyses of variance were not violated.

## 3. Results

### 3.1 Individual subject noise averaging

For each participant, we generated a trustworthy face (CI, classification image see Methods). We repeated this procedure for attractiveness judgements. The averages for selected noise, for trustworthiness and attractiveness for Asian and European descent participants when evaluating both Asian and European descent faces are shown in **Fig 2**. **Fig 2A** shows classification images of trustworthy (left) and untrustworthy (right) European descent faces (signal-to-noise ratio = 1) generated by one representative French participant and **Fig 2B** shows the classification images of attractive (left) and unattractive (right) Asian faces (signal-to-noise ratio = 1) generated by a Chinese participant.

### 3.2 Group-level noise averaging

**Fig 3:**
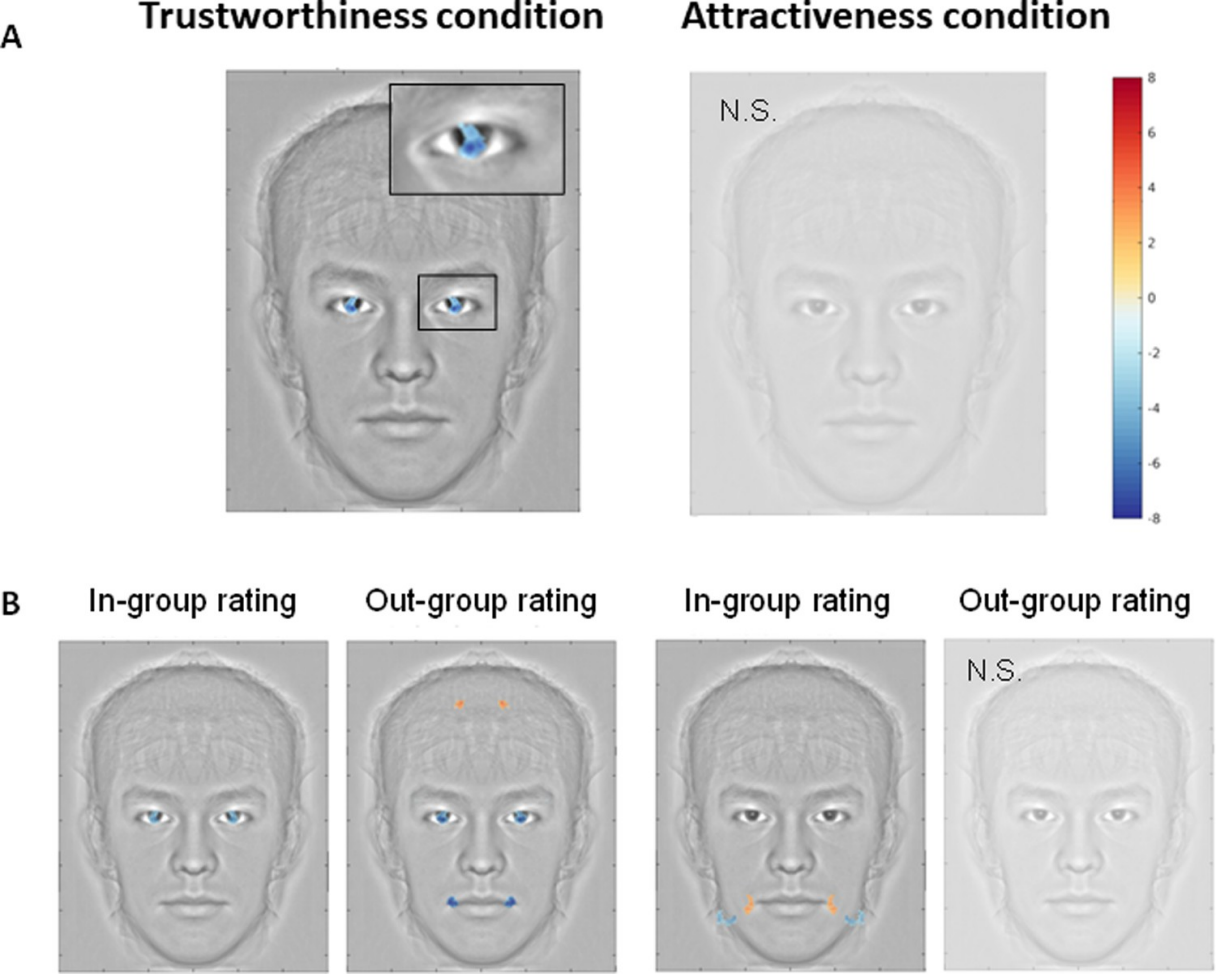
Conjunction analyses. Conjunction analyses reveal, among all participants’ classification images, which facial cues were consistently modulated by a trustworthiness or an attractiveness judgment. For each task, significantly modulated pixels were identified using a cluster-based permutation test (p<0.05, FWER corrected for multiple comparisons). Then, only pixels that were significant for all considered tasks were conserved and the minimum observed z-score were displayed. A positive z-score indicates that the area is consistently lightened (i.e. subjects selected the picture in which this region contains more light pixels) while a negative z-score indicates that the area is consistently darkened (subjects selected the picture in which this region contains more dark pixels). **(A)** Conjunction analysis between both populations (Chinese, French) and two stimuli types (Asian, European descent) for the trustworthiness (left) and attractiveness (right) judgments. Results showed a clear and significant pattern of classification across populations and faces only for trustworthiness judgments. For this condition, Chinese and French participants darkened selectively the face area within the pupil-iris area of the eyes to make the face more trustworthy (see zoom). No equivalent selective area overlapped for attractiveness judgements. (**B)** Partial conjunction analyses for in-group (Chines rating Asian faces + French rating European descent faces) and out-group (Chinese rating European descent faces + French rating Asian faces) ratings for a trustworthiness (left) and an attractiveness (right) judgment. For trustworthiness in-group ratings, participants darkened the eyes area; while for out-group rating participants not only darkened this area, but also the extremity of the mouth, as though to make the faces more smiley. This result suggests that the smile is not the most reliable cue for a trustworthiness judgment in our everyday social environment. However, the smile remains an important cue for rapid and automatic evaluation of strangers. Concerning attractiveness, for in-group ratings, participants darkened the upper part of the jaw and lightened the extremity of the mouth, thus forming a chiseled jawline for the attractive face and a more rounded appearance for the non-attractive picture. However, for out-group ratings, no face region emerged as significant.

In a second step, we averaged the CIs across participants (See Methods section for details). By doing so, pixels that are consistently modulated across the population are highlighted. First, we calculated an average of 80 CIs obtained from the 40 French participants who trust judgments [and attractiveness for the control condition] on European and Asian descent faces, and an average of 80 CIs obtained from the 40 Chinese participants who made trust judgments on European and Asian descent faces (**S2 Fig in S1 File**). Then, we computed, a group-level average CI of 160 images of the two types of stimuli (Asian and European descent faces) from each of the 80 participants’ CIs (40 French, 40 Chinese) and of each judgment (trustworthiness and attractiveness) (see **Fig 2**). Grand CI averages for trust and attractiveness were calculated by combining the individual CIs obtained for the participants and images from both ethnic groups (Fig 3C and 3D, respectively). We also calculated the mean CIs separately for the French and Chinese participants (**S2 Fig in S1 File**). Interestingly, few pixels were consistently modulated by observer and stimulus ethnicity, suggesting that most of the parameters of our internal representations are not cross-cultural but relatively culture or even subject-specific. As can be seen in **Fig 2C**, the average cross-cultural trust CIs are characterized by lighter areas in the mouth and eyebrow regions and darker areas in the eye and hair regions. As can be seen in **Fig 2D**, attractiveness related CIs are characterized by light areas around the nose and the mouth, and dark areas on the forehead, nose and eyebrow.

### 3.3 Conjunction analysis

To identify consistently modulated pixels in different populations and stimuli for trustworthiness and attractiveness, we performed a conjunction analysis and a cluster-based permutation test, FWER corrected for multiple comparisons (p<0.05). The conjunction analysis on grand average CIs across subjects and stimulus ethnicity showed a clear and significant pattern of classification for trust judgements but not for attractiveness judgments (See **Fig 3A)**. For trustworthiness, during face selection the Chinese and French perceivers generated images with higher pupil-sclera contrast. On the other hand, for attractiveness judgments, no area overlapped across cultures, meaning that there are no universal facial features contributing to attractive faces.

**Fig 3B** shows the partial conjunction analysis, distinguishing between in-group and out-group faces judgements. The in-group conjunction analysis, displayed in **Fig 3B** (i.e., Chinese rating Asian faces + French rating European descent faces), shows that when perceivers were asked to choose trustworthy faces from among the faces in the same group, they selected faces with higher contrast between the iris and sclera, thus focusing only on the eye region. On the other hand, the lighter end of the lips and darker upper jaw increased participants’ perceived attractiveness to faces from the same culture.

Next, outgroup conjunction analyses, shown in **Fig 3B** (i.e., Chinese rating European descent faces + French rating Asian faces) demonstrate that the darker eyes region, darker lip tips and lighter forehead increased the perception of trustworthiness of outgroup faces. In contrast, when participants selected attractive faces from a different group of faces, no common areas survived statistical analysis between participants.

To summarize, high-contrast eyes were common to both in-group and out-group trustworthy faces but no common features emerged for attractiveness. See **Table 1** for a summary of the results.

**Table 1:** Results summary.

	Trustworthiness condition	Attractiveness condition
**Ingroup rating**	Eyes area darkened	Upper part of the jaw darkened and extremity of the mouth lightened
**Outgroup rating**	Eyes and mouth area darkened	Not significant

### 3.4 Parametric analysis on eye ROI

First, we defined an eye region of interest (ROI) based on the cluster that emerged from the conjunction analysis (See **S3 Fig in S1 File**). Then, an eye contrast-based score was calculated for each individual CI as the average intensity extracted from this ROI.

Because our participants were both male and female, but we used only male faces in our stimulus set, we further tested whether the eye ROI contrast score varied as a function of participant gender (See **S3 Fig in S1 File**). We applied a two-way analysis of variance (ANOVA) with gender (male vs female) and population (French vs Chinese) as factors on the ROI contrast score. As predicted by the conjunction analysis, there was no significant main effect of population (F_1,76_ = 0.4, p>0.530), but there was a significant main effect of gender (F_1,76_ = 8.44, p = 0.005) without a significant interaction effect (gender X population) was found (F_1,76_≈0, p>0.97). Post-hoc analysis with the Bonferroni correction confirmed the high effect of trust on the eye area regardless of the sex of the subject (one simple t-test, N = 40 male, p<6.34x10^-6^, N = 40 female, p<8.67x10^-11^) but as revealed by the ANOVA the observed eye-effect was slightly higher for female than for male (two simple t-test, N = 80, p = 0.004).

## 4. Discussion

Here, we studied how humans construct an internal representation of trust using a reverse correlation task in Chinese and French participants. Participants were asked to select the face that they perceived as more trustworthy from two noisy versions of the same face. As expected, we found that there are specific areas of the face that individuals from different cultures use to build an internal representation of trust. The specific facial feature for trust-that emerged as a “universal” cue was high contrast eyes characterized by large dark pupils. Chinese and French participants *implicitly* darkened the eye region of Asian and European descent faces, to make the face more trustworthy. As many studies have shown, the eye region conveys unique information for understanding others’ intentions, a critical ability important for adaptive social interaction [25, 26]. Our results demonstrated that trust decision-making, regardless of face familiarity or ethnic group, relies on the eyes perception, which can be regarded as the most salient social feature informing about the internal state of others’. It is indeed known that eye gaze has a crucial role in detecting the focus of attention of other individuals and in inferring their goals [27–29]. From a developmental point of view, the attraction of the eye area appears spontaneously in two month-old children, and its absence is associated with severe pathologies [30]. For instance, individuals who show difficulties looking into the eyes of others might have clinical conditions such as schizophrenia [31], autism [32] or focal brain damage [33]. Eyes attraction, appears to be a deeply rooted evolutionary behavior as monkeys show interest in the facial area of their conspecifics, and the eye region in particular, during social interactions [34–36] or spontaneously direct their attention to human eyes during passive observation [10].

The fact that the eyes are the only facial region to stand out when assessing trust in different cultures may depend on their unique morphological configuration. For example, human eyes have a large white sclera surrounding the pupil-iris area, which facilitates discrimination of gaze direction and allows individuals to guess what the other is looking at and their intentions [37]. The contrast of the white sclera in humans is unique among primates and is thought to have coevolved with the social nature of humans. The sclera of most primate eyes is brown with only a few species having light brown or partially white sclera. In infant macaques, the sclera is less pigmented than adults. A darker sclera in adult macaques, may offer the advantage of looking out of the corner of the eye, without explicit eye contact that could be associated with a potential threat [38]. Determining the exact direction of another parson’s attention is difficult to estimate when there is no contrast between the sclera and the iris. In humans, the interpretation of the direction of gaze is facilitated by the morphology of the eyes and particularly by the discrimination between the sclera and the iris [37].

A recent study showed that pupil mimicry is related to trusting behavior in humans [39]. Taking an interest in another’s pupils and synchronizing with their changes provides an assessment of trustworthiness. The authors claimed that, when humans unconsciously mimic another person’s pupil dilation, they also experience a resonance of that person’s inner state, signaling mutual interest and liking. This process could enable rapid trust-related decisions in interactions with strangers. In our study, pupils played a key role, as participants’ sense of trust was stronger for faces having darker pupil-iris areas.

Another line of research supports the view that iris clarity may play a crucial role. Kleisner et al. [40] for instance found that faces with brown-eyes were perceived as more trustworthy than blue-eyed faces. This is in consistent with the phylogenetic view that brown eyes developed first during human evolution, while blue eyes appeared as a result of a genetic mutation affecting the OCA2 gene [41]. In addition, brown-eyed people are perceived as happier and less shy than blue-eyed individuals [42, 43], consistent with findings showing that trustworthy faces are linked to positive emotions [44].

We observed that when participants were asked to judge the trustworthiness of an out-group (cross-cultural) compared to an in-group (intra-cultural), additional cues were required. Specifically, when participants rated in-group trust, only the eyes appeared as a mandatory region for constructing an in-group internal representation of trust, whereas cues from the mouth region were also involved in out-group trust. This result suggests that when assessing trust between faces belonging to a different ethnic group, subjects focus on additional areas, as if additional information must be gathered for the individual to make a decision. It is important to note that the nature of the contribution of this additional signal is consistent across participants and cultures. As previously described, the mouth, along with the eyes, convey critical pieces of information for humans to decide whether to trust or not another individual [14]. Indeed, the mouth region plays a critical role according to the emotion overgeneralization hypothesis of trust. Individuals would select neutral faces that implicitly resemble happy faces as trustworthy. Given that previous reports have indicated that emotional expressions are perceived differently from outgroup to ingroup [45], we extend this result by demonstrating that individuals can look for additional evidence by considering the emotional expression of the mouth region of outgroup faces when attempting to infer trustworthiness.

In this study, when assessing trust, the within-cultural effects (**S2 Fig in S1 File**) show that the corner of the mouth, the smile, is a relevant region for all groups except the French perceivers. French perceivers might think that the signal transmitted by a smile can be false, but only when judging faces from their own culture. Previous studies have shown that culture can influence how people perceive the honesty of smiles in others [46]. These results are presented for illustrative purposes but, due to the configuration of the task they should be taken with caution. Indeed, the observed modulation of intra-cultural trust judgement (i.e., Chinese individuals judging Asian faces, or French individuals judging European descent faces), could be due to the different stimuli used (Asian vs European descent faces).

In contrast to trust, when individuals rate ingroup attractiveness, only culture-specific cues appear, such as the mouth and the upper jaw, which do not appear when evaluating outgroup attractiveness. Modulating this area allowed participants to make the bone structures of the mandibular section more visible for attractive faces and more blurred, resembling a rounded face, for the unattractive ones.

The absence of universal or culturally unrelated attractiveness cues suggest that judgements about attractiveness, at least among adults, are limited by learning, education, cultural imprinting or expertise and cannot be extended to other cultures without experience. Therefore, the fact that no area appeared consistently modulated to produce a judgment of attractiveness among faces from another culture is consistent with psychological models such as the *other-race-effect* [47] and imprinting [23]. According to these models, individuals do not construct a stereotypical representation of what is attractive in another culture.

Moreover, previous works have shown that attractiveness is based on face symmetry and stereotypy [[23] for a review]. Given that our faces were homogeneous (in terms of symmetry and average), this could have reduced the possibility of obtaining a clear divergence between attractive and unattractive faces.

Trust is the basis of human societies, cooperation and helpful behaviors. It is fundamental to understand how people construct internal representations of trust across cultures. This is of particular relevance in modern societies where, thanks to mass migration and globalization, individuals increasingly interact with people from different cultures. Whether different cultures have unique ways of perceiving, establishing, and maintaining trust was previously unknown. Here, we show that the pupil-iris area of the eye plays a fundamental role in establishing trust independent of cultural mores.

## Supporting information

S1 File (DOCX)
