# Supplementary material for: Culture-free perceptual invariant for trustworthiness
Source: PLoS One. 2022 Feb 10;17(2):e0263348. doi: 10.1371/journal.pone.0263348 (PMC8830731; doi:10.1371/journal.pone.0263348)
Supplement: S1 File — (DOCX) [file pone.0263348.s001.docx]

**Supplementary methods & results**

**Figure S1**


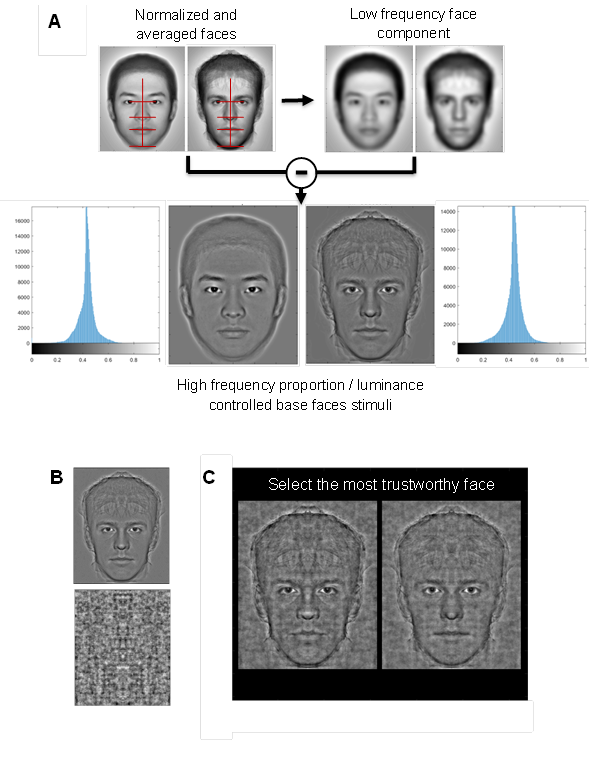


**Figure S1.** Stimuli construction and experiment design. (A) Images were created by averaging symmetrized and proportionally controlled Asian and European faces to create a stereotyped face for each ethnic group. Then, low frequency components of the images were removed and low-level properties of the two reference stimuli were controlled with histogram equalization. (B) Example of a noise mask generated from the reference stimuli. At each trial, a noise mask was generated by 1) randomizing the phase of the image via fast Fourier transformation; 2) symmetrizing the noise pattern by averaging the phase randomized picture with its flipped left-right counterpart. In this way, generated noise patterns have the same frequency content, luminance distribution and symmetric property of the original face picture. (C) During the reverse correlation task, on each trial, a noise pattern was generated and added (left) or removed (right) from the original face stimulus (signal/noise ratio=1). Then, participants have to decide which noisy picture looked the most trustworthy/attractive. Participants were asked to respond based on a first impression judgment and the two images were presented on the screen until the response.

From this intra-cultural results, it appears that the eyes, the corners of the mouth and the eyebrows relate to trustworthiness for Chinese perceivers, regardless of whether the face is Asian or European. The eyes and the mouth are important for trustworthiness judgments, except in the case of French perceivers judging ingroup faces (where only the eye region is important). Moreover, French perceivers have significant pixels for the attractiveness of the outgroup faces, but the Chinese do not.

**Figure S2**

*
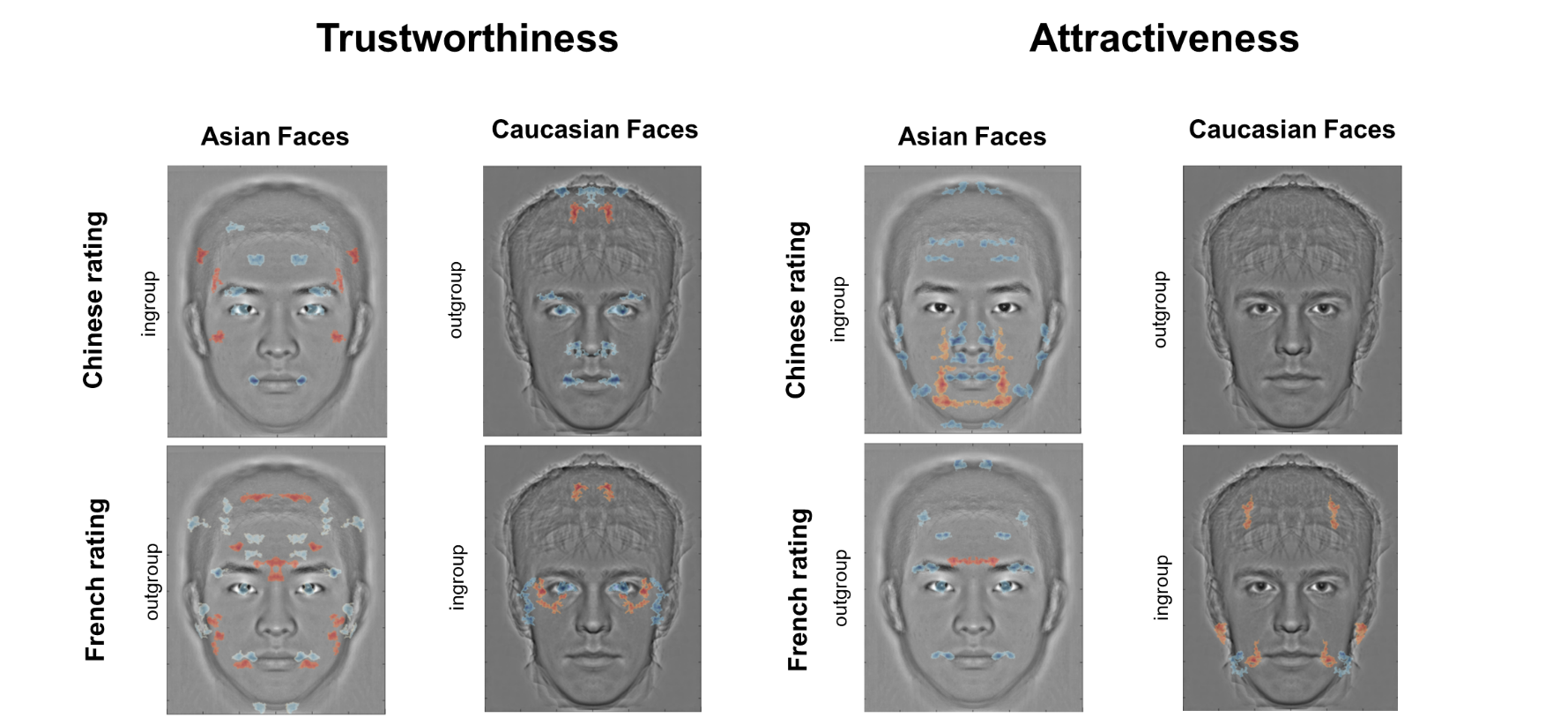
*

**Figure S2*.*** Mean classification images.

Ingroup and outgroup rating for trustworthiness and attractiveness judgments. Each face has been created averaging 80 CIs obtained from the 40 Chinese and 40 French participants that made trustworthiness [attractiveness on the left] judgments of European and Asian faces.

**Figure S3**

**
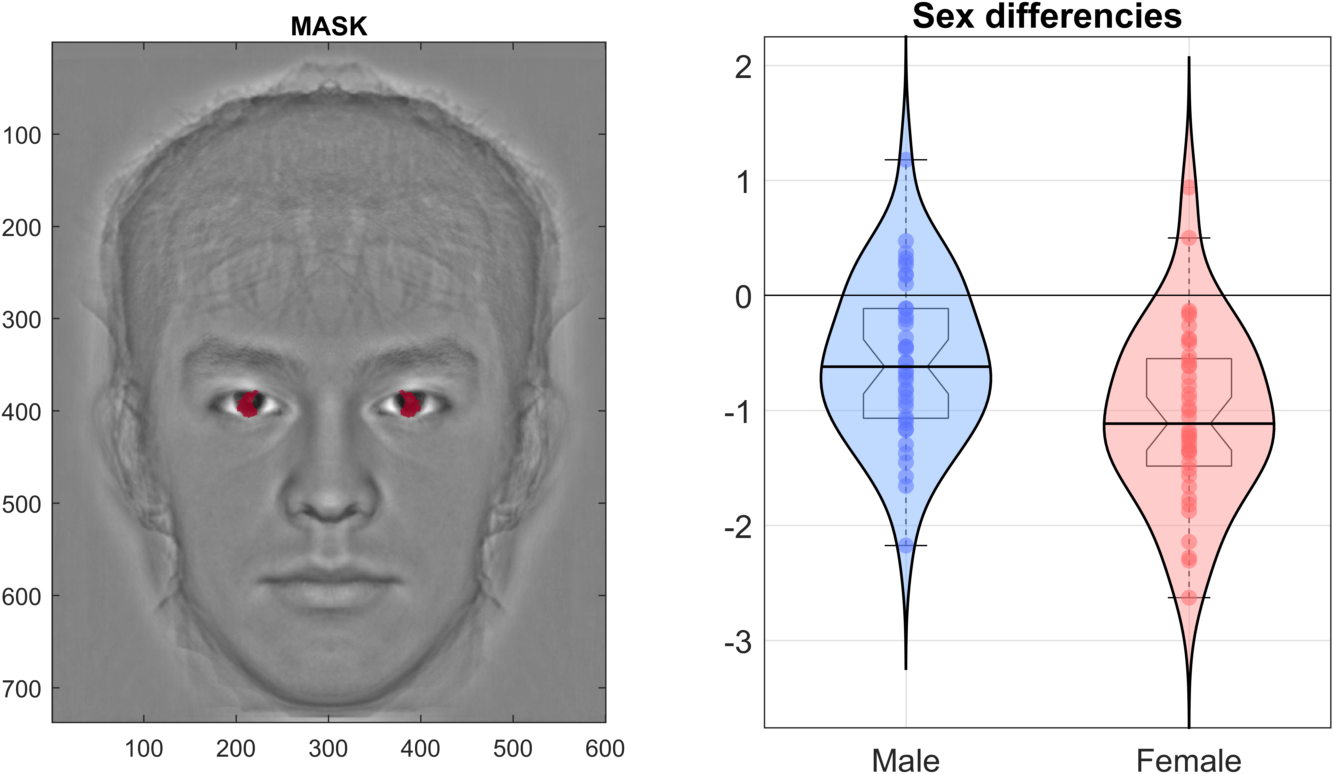
**

**Figure S3**. Eyes cluster mask used to define the face region of interest for testing the effect of gender on trustworthiness.
